# Fatal Disseminated *Fusarium* Infection in a Human Immunodeficiency Virus Positive Patient

**DOI:** 10.1155/2013/379320

**Published:** 2013-05-13

**Authors:** Ashwini K. Esnakula, Irorere Summers, Tammey J. Naab

**Affiliations:** Department of Pathology, Howard University Hospital, 2041 Georgia Avenue NW, Washington, DC 20060, USA

## Abstract

Systemic mycotic infections have been increasing in incidence in immunocompromised patients. Although yeasts are most often isolated, opportunistic fungal infections may also be caused by filamentous fungi, including *Aspergillus* and *Fusarium*. Like *Aspergillus*, *Fusarium* is angioinvasive with an ability to disseminate widely. Disseminated fusariosis is most commonly linked to prolonged neutropenia. Disseminated infections due to *Fusarium* are rare in Human Immunodeficiency Virus (HIV) positive patients but have been reported in HIV positive patients with neutropenia and lymphoma. We describe an HIV positive patient without neutropenia, skin lesions, or concomitant malignancy, who developed fatal disseminated infection with possible endocarditis due to *Fusarium solani*. Early identification of *Fusarium* is important because of its high level of resistance to several antifungal drugs, with response often requiring combination therapy.

## 1. Introduction

Systemic mycoses are most often caused by yeasts in immunocompromised hosts. *Candida* and *Cryptococcus *are the most frequent isolates. Recently, filamentous fungi (molds) have been increasingly identified in disseminated and sometimes fatal opportunistic infections in Human Immunodeficiency Virus (HIV) positive patients with AIDS, patients with hematologic malignancies, and hematopoietic stem cell transplant recipients. This increase in filamentous fungal infections has been attributed to antifungal prophylaxes used to prevent yeast infections [1]. The most commonly isolated mold is *Aspergillus*. *Fusarium*, *Scedosporium,* and *Penicillium* and the aseptate Zygomycetes have also been increasing in incidence. Neutropenia, T-cell deficiency, and high dose corticosteroid therapy are important risk factors for developing mold infections [2]. For unknown reasons, *Fusarium* infections are rare in HIV positive patients [3]. We describe an AIDS patient without neutropenia developing fatal disseminated disease with possible infective endocarditis due to *Fusarium solani. *This is the first case report of fatal disseminated *Fusarium* infection in an AIDS patient without neutropenia.

## 2. Case Description

**Figure 1:**
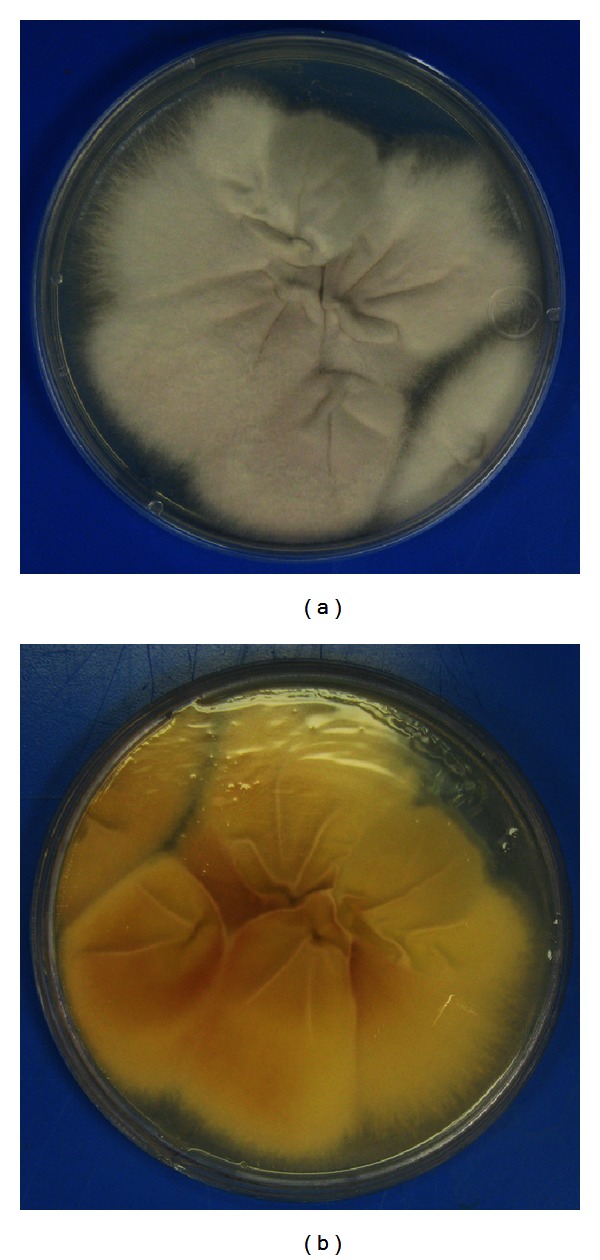
Sabouraud's dextrose agar without cycloheximide showing cottony white mold (a) with cream-colored reverse (b).

**Figure 2:**
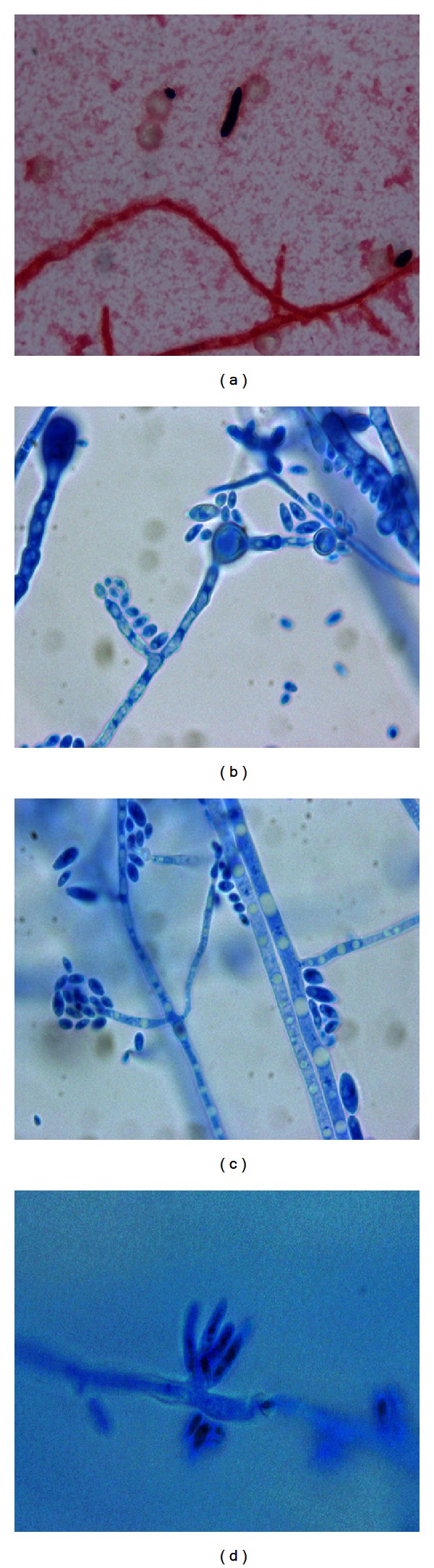
Gram stain of blood culture showing gram negative septated hyphae and gram positive microconidium and macroconidium (a). Lactophenol cotton blue slide preparations (LPCB) showing septated hyphae with intercalary chlamydoconidia (b) (X1000). Septated hyphae with straight simple thin long phialide bearing oval microconidia in a “diphtheroidal” pattern (c) (LPCB, X1000). Macroconidia in clusters, arising on a small conidiophore (d) (LPCB, X1000).

A 44-year-old African-American HIV positive male with a past medical history of AIDS, dialysis-dependent end-stage renal disease due to HIV associated nephropathy, chronic hepatitis C, and seizure disorder was admitted with chief complaint of intractable diarrhea of one month's duration. The CD4 T-lymphocyte count was 64/*μ*L, and the viral load was 500,000 copies/mL. Cytomegalovirus (CMV) colitis was diagnosed based on positive blood CMV by PCR, and intravenous ganciclovir therapy was administered. Highly Active Antiretroviral Therapy (HAART) was also initiated for the first time; high dose corticosteroids were started because of the risk of Immune Reconstitution Inflammatory Syndrome (IRIS). During his hospital course, the diarrhea subsided, but he developed persistent fever. Chest X-ray and computed tomography [2] scan showed diffuse bilateral infiltrates with areas of consolidation; a diagnosis of hospital-acquired pneumonia was made. The CBC at this point showed WBC count of 11.8 × 10^3^/mm^3^ with 10.4 × 10^3^/mm^3^ neutrophils. The patient subsequently developed severe hypoxia requiring intubation and admission to the medical intensive care unit. The blood cultures drawn at this point were positive after three days of incubation. Gram stain of the cultures revealed septated hyphae with occasional branching at a narrow angle (Figure 2(a)). Fungemia was diagnosed, and amphotericin B deoxycholate was instituted. A cottony white mold (Figure 1(a)) with cream-colored reverse (Figure 1(b)) grew at 4 days on Sabouraud's dextrose agar without cycloheximide. Lactophenol cotton blue preparation revealed hyaline, septated hyphae with numerous intercalary smooth thick-walled chlamydoconidia arranged singly and in pairs (Figure 2(b)). Clusters of oval, unicellular microconidia, measuring 5–8 × 2–4 *μ*m, were arranged in a diphtheroidal pattern at the ends of long, thin, straight, and single phialides (Figure 2(c)). Scattered clusters of crescent-shaped septated macroconidia, measuring 32–35 × 4–6 *μ*m, were present at the ends of short conidiophores (Figure 2(d)). Based on these morphologic features, the mold was consistent with *Fusarium solani*. A central venous catheter was removed since it was suspected to be the source of infection. An echocardiogram showed a right atrial thrombus suggestive of infective endocarditis. The patient developed Disseminated Intravascular Coagulation (DIC) and died six weeks after admission.

## 3. Discussion

*Fusarium *is a ubiquitous, thermally monomorphic saprophyte, found in soil, on plants, associated with foreign bodies (contact lenses, vascular catheters), and in aquatic habitats including hospital plumbing systems [4]. It is an economically significant plant pathogen and causes infections in animals and humans. *Fusarium *is one of the hyalohyphomycetes. The tissue forms are characterized by hyaline, pale, and nonpigmented hyphae, that demonstrate narrow-angle branching, parallel walls, and septa. The hyphae are clinically indistinguishable in tissue from *Aspergillus. *Like *Aspergillus, *they invade blood vessels walls leading to thrombosis and necrosis of surrounding tissue [5]. The three most common *Fusarium *spp. associated with human infections are *Fusarium solani *(50%)*, Fusarium oxysporum *(20%), and *Fusarium verticillioides *(formerly *F. moniliforme*) (20%) [6].

*Fusarium *causes a wide spectrum of infections in humans. Host immunity plays a role in the severity of the disease. Single organ involvement and most foreign-body associated fusariosis are observed in patients with normal immunity. The most common localized infections in normal hosts are onychomycosis and keratitis in soft contact lens wearers. Locally invasive cutaneous disease is occasionally observed in immunocompetent patients with wounds or burns. Less common infections in hosts with intact immunity are endophthalmitis complicating advanced keratitis, myositis, arthritis, and peritonitis in patients receiving chronic ambulatory peritoneal dialysis. 

At the highest risk for disseminated fusariosis are immunocompromised patients with hematologic malignancies on cytotoxic therapy and transplant recipients. The most important predisposing factor in patients with leukemia is prolonged neutropenia (absolute neutrophil count < 1000 cells/*μ*L). Defense against mold infections is closely linked to innate immunity mediated by neutrophils and macrophages. Neutrophils are the most critical element in fighting *Fusarium *infections because they are able to destroy fungal hyphae by producing NADPH oxidase; macrophages block germination of conidia [7]. Allogeneic HLA-mismatched Hematologic Stem Cell Transplant (HSCT) recipients have a high incidence of fatal *Fusarium *infection due to T-cell deficiency associated with graft-versus-host-disease and high dose corticosteroid therapy [6, 8, 9]. 

Disseminated disease constitutes 70% of *Fusarium *infections in immunocompromised patients. Skin is commonly involved with the most frequent presentation being multiple tender, and erythematous nodules having black centers due to central necrosis. Angioinvasion by *Fusarium *hyphae with thrombosis and infarct accounts for the ecthyma gangrenosum-like appearance [10]. Cutaneous lesions and fungemia represent the most frequent presentation of disseminated infection with disseminated disease being linked to central venous catheters. When skin lesions represent a manifestation of fungemia, mortality is 100%. Lung involvement, characterized by nonspecific bilateral infiltrates, sometimes cavitary, has high mortality and often occurs with disseminated infection [6]. Other organs involved in disseminated disease include sinuses, liver, spleen, kidneys, and rarely brain. 

For unknown reasons, disseminated *Fusarium* infections are rare in HIV positive patients. A review of 294 patients published in 2005 identified only two cases of disseminated *Fusarium* infections in HIV positive patients. Of these two HIV positive patients, one had a disseminated *Fusarium oxysporum* infection related to an infected port-a-catheter, which was successfully treated with liposomal amphotericin B [11]. The other HIV positive patient had neutropenia secondary to chemotherapy for high grade non-Hodgkin lymphoma [12]. No case of disseminated *Fusarium *infection has been reported since 2005. Our patient represents the first report of fatal disseminated *Fusarium* infection in a nonneutropenic HIV positive patient. Severe prolonged neutropenia, the most significant predisposing risk factor, is uncommon in HIV positive patients and might explain why disseminated *Fusarium* infections are rarely encountered. T-cell deficiency is the other major risk factor. In general, T-cell deficiency has become less common in HIV positive patients because of institution of HAART at the asymptomatic stage when T cells are within normal range. Possibly, disseminated filamentous fungal infections are less likely to develop due to preservation of T-cell function.

Our patient had several factors which increased his risk of disseminated fungal infection. Having never received HAART prior to this admission, he had significant T-cell deficiency. Due to high viral load, his neutrophils, although adequate in number, were dysfunctional since the HIV impairs oxidative respiratory burst and leads to decreased chemotaxis [13]. Our patient had end-stage renal disease requiring hemodialysis, which is associated with loss of T-cell function, neutrophil dysfunction due to bioincompatible dialysis membranes, and poor response to phagocytic cytokines [14]. Advanced chronic kidney disease is associated with high serum levels of interleukin-2 receptor, which is linked to T-cell dysfunction [15]. Our patient was at high risk for Immune Reconstitution Inflammatory Syndrome (IRIS) because of the low CD4 count, late administration of HAART in a naïve patient, and coexistent CMV colitis. An important deleterious effect of IRIS is related to upregulation of Th17, a subset of T cells in the mucosa associated lymphoid tissue of the gastrointestinal tract; Th17 cells release many proinflammatory cytokines leading to tissue damage in the host and increased risk of disseminated opportunistic infection, in our case, due to *Fusarium solani* [2, 16, 17]. 

Diagnosis of *Fusarium* requires culture with identification of characteristic morphologic features. Any involved tissue, especially skin, should be biopsied and sent for culture. Blood cultures are often positive (up to 77%) when compared with *Aspergillus*. *Fusarium* grows rapidly on most mycologic media without cycloheximide. The blood culture isolates in our case produced white, powdery colonies with reverse cream color in 4 days on Sabouraud's dextrose agar without cycloheximide. *Fusarium *genus specific septated, crescent-shaped macroconidia were identified [18]. In our case, the production of long, straight, and thin phialides and the presence of many hyaline intercalary chlamydoconidia, sometimes in pairs, confirmed the identification of *F. solani *[18]. In cases in which diagnostic morphology is not apparent, *s*pecies identification may be confirmed with nested PCR molecular assay, which amplifies internal transcribed space regions between 18S rDNA and 28S rDNA [1]. Mass spectroscopy using matrix-assisted laser desorption ionization-time of flight (MALDI-TOF) may soon be available allowing for rapid identification of fungi (within one hour), but no validated protocol has been developed.

Serological assays, including galactomannan and 1,3-*β*-D glucan, have some value in diagnosing *Fusarium *infection. The 1,3-*β*-D glucan assay is often positive in invasive *Fusarium* infections but is also detectable in fungal infections caused by *Candida, Aspergillus, *or *Trichosporon.* Galactomannan is usually negative in invasive *Fusarium* infections, so that positive 1,3-*β*-D glucan assay and negative galactomannan make *Fusarium* a serious consideration [1]. 

When compared to other filamentous fungal pathogens, *Fusarium* demonstrates greater resistance to many antifungal drugs with intrinsic resistance to older azoles (e.g., fluconazole, itraconazole) and echinocandins (e.g., caspofungin, micafungin) and variable resistance to triazoles and amphotericin B. High dose liposomal or lipid complex amphotericin B is the preferred treatment of choice in disseminated infections [1]. Case reports have shown increased survival by combining liposomal amphotericin B with voriconazole or posaconazole [1, 2, 19, 20].

## 4. Conclusions 

Fatal disseminated infections due to filamentous fungi are increasing in incidence in immunocompromised hosts. Major risk factors are prolonged, profound neutropenia in patients with hematologic malignancies and HSCT recipients with T-cell deficiency on corticosteroids. Although rare in HIV positive/AIDS patients, disseminated fusariosis can occur, especially if cumulative factors exist, leading to neutrophil dysfunction and T-cell abnormalities. Prompt diagnosis of disseminated *Fusarium *infection is recommended due to innate resistance to multiple commonly used antifungal drugs. Adjunctive measures, including surgical debridement and administration of granulocyte transfusions and/or granulocyte-macrophage colony-stimulating factor (GM-CSF) leading to normalization of the neutrophil count, have been associated with some improvement in outcome in immunocompromised patients. 

## References

[1] Muhammed M, Coleman JJ, Carneiro HA, Mylonakis E (2011). The challenge of managing fusariosis. *Virulence*.

[2] Perfect JR (2012). The impact of the host on fungal infections. *American Journal of Medicine*.

[3] Galimberti R, Torre AC, Baztan MC, Rodriguez-Chiappetta F (2012). Emerging systemic fungal infections. *Clinics in Dermatology*.

[4] Short DP, O'Donnell K, Zhang N, Juba JH, Geiser DM (2011). Widespread occurrence of diverse human pathogenic types of the fungus Fusarium detected in plumbing drains. *Journal of Clinical Microbiology*.

[5] Dignani MC, Anaissie E (2004). Human fusariosis. *Clinical Microbiology and Infection*.

[6] Nucci M, Anaissie E (2007). Fusarium infections in immunocompromised patients. *Clinical Microbiology Reviews*.

[7] Leal SM, Vareechon C, Cowden S (2012). Fungal antioxidant pathways promote survival against neutrophils during infection. *The Journal of Clinical Investigation*.

[8] Campo M, Lewis RE, Kontoyiannis DP (2010). Invasive fusariosis in patients with hematologic malignancies at a cancer center: 1998–2009. *Journal of Infection*.

[9] Park BJ, Pappas PG, Wannemuehler KA (2011). Invasive non-Aspergillus mold infections in transplant recipients, United States, 2001–2006. *Emerging Infectious Diseases*.

[10] Nucci M, Anaissie E (2002). Cutaneous infection by Fusarium species in healthy and immunocompromised hosts: implications for diagnosis and management. *Clinical Infectious Diseases*.

[11] Eljaschewitsch J, Sandfort J, Tintelnot K, Horbach I, Ruf B (1996). Port-a-cath-related Fusarium oxysporum infection in an HIV-infected patient: treatment with liposomal amphotericin B. *Mycoses*.

[12] Guarro J, Nucci M, Akiti T, Gene J (2000). Mixed infection caused by two species of Fusarium in a human immunodeficiency virus-positive patient. *Journal of Clinical Microbiology*.

[13] Hadad N, Levy R, Schlaeffer F, Riesenberg K (2007). Direct effect of human immunodeficiency virus protease inhibitors on neutrophil function and apoptosis via calpain inhibition. *Clinical and Vaccine Immunology*.

[14] de Marie S (1993). Diseases and drug-related interventions affecting host defence. *European Journal of Clinical Microbiology & Infectious Diseases*.

[15] Donati D, Degiannis D, Homer L, Gastaldi L, Raskova J, Raska K (1991). Immune deficiency in uremia: interleukin-2 production and responsiveness and interleukin-2 receptor expression and release. *Nephron*.

[16] Grant PM, Komarow L, Andersen J (2010). Risk factor analyses for immune reconstitution inflammatory syndrome in a randomized study of early versus deferred ART during an opportunistic infection. *PloS One*.

[17] Klatt NR, Brenchley JM (2010). Th17 cell dynamics in HIV infection. *Current Opinion in HIV and AIDS*.

[18] Sutton DA, Fothergill AW, Rinaldi MG (1998). *Guide to Clinically Significant Fungi*.

[19] Ho DY, Lee JD, Rosso F, Montoya JG (2007). Treating disseminated fusariosis: amphotericin B, voriconazole or both?. *Mycoses*.

[20] Vagace JM, Sanz-Rodriguez C, Casado MS (2007). Resolution of disseminated fusariosis in a child with acute leukemia treated with combined antifungal therapy: a case report. *BMC Infectious Diseases*.

